# Cultured Meat and Its Acceptability in Muslim Societies: A Narrative Perspective on Halal Perspectives and Regulatory Challenges

**DOI:** 10.3390/foods15081288

**Published:** 2026-04-09

**Authors:** Randah M. Alqurashi, Dominika Sikora, Piotr Rzymski, Barbara Poniedziałek

**Affiliations:** 1 Department of Food and Nutrition Sciences, College of Agriculture and Food Sciences, King Faisal University, Al-Ahsa 31982, Saudi Arabia; 2 Department of Environmental Medicine, Poznan University of Medical Sciences, 60-806 Poznan, Poland; 3 Doctoral School, Poznan University of Medical Sciences, Fredry St. 10, 61-701 Poznan, Poland

**Keywords:** cultured meat, Islam, novel food, halal certification, food ethics, sustainable food systems, biotechnology, religion

## Abstract

Cultured meat holds the potential to reduce environmental impacts and offer ethical advantages while replicating the nutritional, taste, and texture attributes of conventional meat. To date, most research on consumer acceptance of meat has focused on European and North American markets. In contrast, Muslim-majority countries remain underexplored, particularly regarding the compatibility of cultured meat with Islamic dietary laws. These societies are experiencing rising meat consumption, and countries such as Saudi Arabia and Malaysia rely heavily on meat imports. This narrative perspective article aims to systematically examine how specific stages of cultured meat production align with, or challenge, Islamic dietary (halal) principles. To this end, we adopt a stage-based analytical approach, mapping key technological steps in cultured meat production onto core requirements of Islamic jurisprudence. To this end, we critically and comprehensively examine the intersection between cultured meat production methods and the Islamic concept of halal, which extends beyond ingredient permissibility to encompass ethical, spiritual, and hygienic dimensions of food production. Key challenges to halal certification include the origin and status of starter cells, whether donor animals were slaughtered according to Islamic law, the permissibility of biopsied tissue, and the use of fetal bovine serum in growth media. The analysis indicates that while halal-compliant cultured meat is scientifically feasible, its adoption remains constrained by unresolved religious interpretations, regulatory fragmentation, and limited availability of halal-certified inputs. We emphasize the need for interdisciplinary collaboration among Islamic scholars, food scientists, certification bodies, and policymakers. From a policy perspective, harmonized halal standards, targeted investment in serum-free and animal-free culture media, and early regulatory engagement with Islamic authorities are essential to facilitate responsible market entry. Therefore, we suggest a multi-level governance and stage-gated halal decision framework for cultured meat. Proactive regulation and open dialogue with religious leaders are vital to ethically introduce cultured meat into Muslim markets, aligning innovation with Islamic values while supporting national sustainability and food security goals.

## 1. Introduction

The global demand for meat continues to rise, and projections suggest this trend will persist [1]. Conventional livestock production undermines environmental sustainability, exacerbates water scarcity, and intensively exploits land resources, particularly in arid regions [2,3]. This challenge is especially pronounced in many Muslim-majority countries, particularly in the Middle East and North Africa, where extreme heat, chronic water scarcity, and limited arable land constrain domestic livestock production and necessitate heavy reliance on imports [4,5,6]. High feed costs, dependence on imported inputs such as soybean meal and maize, and the need for energy-intensive cooling infrastructure further increase production vulnerability and economic volatility [7,8,9]. Recent geopolitical tensions and conflicts in the region have further destabilized supply chains, affecting the availability of essential inputs and amplifying price fluctuations, particularly in the meat sector [10,11]. In addition to environmental pressures, the livestock sector is associated with systemic risks, including vulnerability to zoonotic and transboundary animal diseases, such as avian influenza or African swine fever, which poses a key threat to the stability of the food market [12,13]. At the same time, animal farming uses antibiotics at levels three times those in human medicine, raising concerns about antimicrobial resistance and its consequences for global public health [14,15]. Foodborne pathogens in meat, including *Salmonella*, *Campylobacter*, and *Escherichia coli*, continue to pose risks for millions of consumers worldwide [16]. Despite these drawbacks, conventional meat remains an important dietary component, providing complete proteins and essential nutrients such as iron (Fe), zinc (Zn), and vitamin B12 [17]. Furthermore, meat is deeply embedded in most culinary traditions and is valued for its distinct organoleptic properties [18]. The combination of environmental, public health, and resource-related challenges associated with conventional meat production highlights the need to explore alternative approaches that could complement or partially replace existing systems while preserving the nutritional and cultural roles of food.

**Figure 1:**
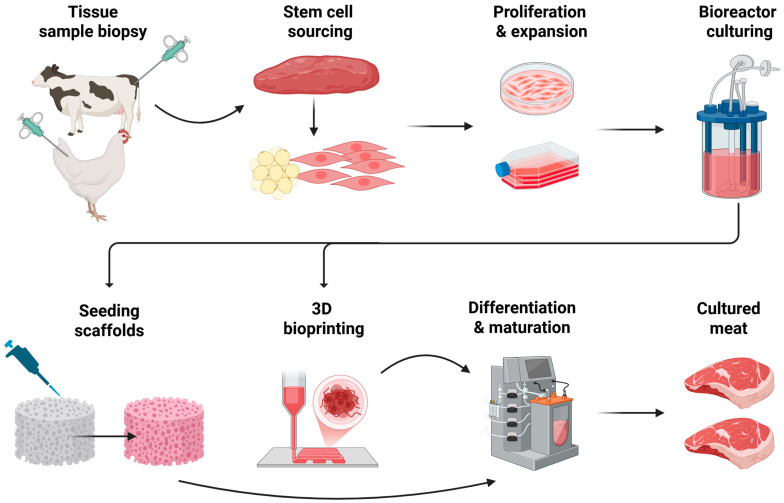
**Schematic presentation of the process of cultivated meat production.** The process begins with a tissue sample biopsy, where a small amount of muscle tissue is collected from a donor animal (e.g., cattle or poultry) without harming the animal. From this biopsy, stem cell sourcing is performed to isolate muscle stem cells and supporting cell types such as adipogenic precursors. These cells then undergo proliferation and expansion in controlled culture conditions, where they are multiplied in Petri dishes or flasks using nutrient-rich growth media. For large-scale production, the expanded cells are transferred to bioreactor culturing, enabling efficient cell growth under tightly regulated parameters such as temperature, oxygen, and nutrient supply. The cells are subsequently seeded onto scaffolds or incorporated into bioinks that provide structural support and mimic the extracellular matrix. Using these materials, 3D bioprinting or scaffold-based assembly is applied to create organized tissue architectures resembling conventional meat. The constructs then undergo differentiation and maturation, during which cells develop into muscle fibers and fat tissue and acquire the desired texture and functionality. The process concludes with the formation of cultivated meat, which is harvested as structured meat products suitable for further processing and consumption. Graph created with BioRender.com.

An emerging alternative to conventional meat is cultured meat (also referred to as cell-cultivated, lab-grown, cell-based, or in vitro meat) [19] (Figure 1). It refers to food products generated through emerging technologies that combine in vitro cell culture techniques with tissue engineering and 3D bioprinting to produce animal muscle tissue under controlled conditions [20]. The process typically begins with the collection of animal tissue via biopsy, followed by the isolation of stem or progenitor cells. Myosatellite cells are most commonly used, but also including stem cells, adipose-derived stem cells, fibroblasts, and endothelial progenitor cells can be employed, depending on the target product. These cells are subsequently expanded and induced to differentiate, after which they are assembled into structured tissue using a range of bioengineering approaches tailored to the desired meat type [21,22,23,24,25].

The first regulatory approval for cultured chicken was granted in 2020 by the Singapore Food Agency, followed by the U.S. Department of Agriculture in 2023 [26,27]. In 2024 and 2025, applications for cultivated foie gras and fatty tissue were submitted to the European Commission for regulatory approval [28,29]. Moreover, in 2025, cultured salmon received pre-market approval from the U.S. Food and Drug Administration [30]. Despite these regulatory advances, some challenges remain in making these products fully available on the market. For example, a recent re-evaluation of cell-cultured chicken meat approved in the United States, based on data submitted to regulatory authorities, found that while the product generally met safety thresholds, notable differences in macronutrient composition and variability across production lots warrant further process optimization and caution against broad generalization across the sector [31]. Nevertheless, both commercialization efforts and research agendas remain largely concentrated in Europe and North America, where consumer interest in cultured meat is often driven by ethical concerns over animal welfare, environmental sustainability, and a broader cultural discourse on meat reduction [32]. By contrast, comparable regulatory assessments, investment strategies, and contextualized governance frameworks are notably underdeveloped in Muslim-majority countries, where additional religious and institutional considerations further shape the pathway to market adoption.

This gap needs to be addressed if one considers that the global halal food market, driven predominantly by Muslim consumers, is expanding rapidly, growing from approximately US$1.27–1.32 trillion in the early 2020s to an estimated US$1.67 trillion by 2025, with halal meat representing one of its largest and fastest-growing segments [33,34,35]. A systematic review further estimates that the halal meat market alone was valued at about US$802 billion in 2021 and is projected to more than double to roughly US$1.66 trillion by 2030, largely due to Muslim population growth, rising incomes, and dietary transitions toward animal protein [34]. These macro-level trends are mirrored in rising per capita meat consumption across many Muslim-majority countries, where intake is projected to increase by 3–17% between 2023 and 2030 in the largest Muslim-population nations, with few exceptions such as Nigeria [33]. Country-level evidence highlights the scale of this shift: in Pakistan, per capita meat consumption increased from 11.7 kg in 2000 to approximately 32 kg by 2020, driven by urbanization, income growth, and changing diets, with earlier projections anticipating levels approaching 47 kg [36]. Similar dynamics are evident in the Middle East and North Africa (MENA) region and Southeast Asia, where population growth, urbanization, and rising disposable incomes have fueled higher meat demand [1]. In wealthier Gulf Cooperation Council states, including Saudi Arabia, United Arab Emirates, Qatar, and Kuwait, per capita meat consumption rose notably between 2010 and 2023, reflecting income-driven dietary shifts toward animal protein [37]. Any of these countries, alongside Southeast Asian nations such as Malaysia, rely heavily on imported meat [38]. Saudi Arabia, for instance, faces persistent food security challenges stemming from its arid climate, limited arable land, and water scarcity, importing approximately 80% of its food supply and remaining vulnerable to global supply chain disruptions and price volatility [39]. In this context, investments in novel food technologies align closely with national development priorities, including Vision 2030 [40]. However, while cultured meat is frequently positioned as a solution to food security, environmental sustainability, and animal welfare concerns, its pathway to adoption in Muslim-majority countries is uniquely shaped by religious and institutional considerations. The absence of formal or harmonized Islamic guidance on the permissibility of cultured meat places halal certification at the center of regulatory uncertainty, constituting a critical barrier to acceptance, governance development, and commercialization in precisely those regions where future demand growth is expected to be strongest.

To strengthen conceptual clarity in addressing this gap, this paper adopts a stage-based analytical framework that maps key cultured meat production steps, cell sourcing, culture media, scaffolding, processing, and certification, onto corresponding halal jurisprudential principles and regulatory checkpoints. In doing so, this paper does not aim to replicate the methods of empirical studies (e.g., consumer surveys) or systematic literature reviews. Rather, as a narrative perspective article, its contribution lies in synthesizing fragmented scientific, theological, and regulatory knowledge into a coherent analytical framework that can guide future research, inform policy discussions, and identify priority areas for empirical investigation. The stage-based model introduced here offers a novel conceptual tool that differs fundamentally from prior efforts that have either focused narrowly on consumer attitudes or addressed halal compatibility in general terms without stage-specific granularity. The aim of this narrative perspective paper is twofold: first, to identify challenges related to cultured meat production in the context of Islamic dietary requirements; second, to propose potential directions and recommendations to facilitate its ethical and religious integration into Muslim societies.

Rather than providing a systematic or exhaustive review, this article adopts an integrative perspective that aligns key stages of cultured meat production, such as cell sourcing, culture media, scaffolds, and processing, with halal jurisprudential principles, certification practices, and regulatory frameworks. By synthesizing scientific workflows with religious and governance considerations, the paper seeks to clarify where technological feasibility intersects with, diverges from, or challenges existing halal certification logic. By addressing regulatory, theological, and technological dimensions in parallel, this perspective paper seeks to clarify unresolved issues and inform policymakers, certification bodies, and industry stakeholders. In doing so, halal compliance is framed not merely as a post hoc certification hurdle, but as a formative design constraint with implications for research priorities, innovation pathways, and market readiness. Although previous studies have examined halal food systems, religious perceptions of biotechnology, and consumer attitudes toward novel foods, these discussions have largely remained fragmented or conceptual. This work advances the field by introducing a stage-based analytical framework linking biotechnological processes with halal decision points, enabling identification of actionable regulatory and technological gaps. Unlike prior conceptual discussions that have addressed cultured meat and Islamic ethics at a general level [41,42], this paper provides a structured, stage-specific analysis that maps key biotechnological steps, cell sourcing, culture media, scaffolding, processing, and certification, onto corresponding halal jurisprudential principles. This approach enables the identification of precise points of compatibility or conflict, thereby moving beyond abstract debates over permissibility toward a framework that can inform both technology development and regulatory design. Realizing these aims is essential to ensure that Muslim-majority countries are not left behind in the global shift toward more sustainable food systems. Addressing religious considerations proactively can support informed policymaking, foster public trust, and facilitate responsible adoption of novel food technologies.

## 2. Halal Certification

The term halal, meaning “permissible” in Arabic, applies to all aspects of a Muslim’s food and dietary practices. Halal certification plays a vital role in ensuring that food products meet Islamic dietary requirements, assuring Muslim consumers that their products align with their religious beliefs [43]. Halal certifiers review all ingredients and processing methods, inspect production facilities and supply chains, and ensure full compliance with Islamic dietary laws. Upon verification, certifying bodies issue official documentation that permits the use of a trademarked halal logo on approved products [44]. Although the halal label is perceived as a reliable indicator, certification standards may vary between countries and organizations. In the context of novel food technologies such as cultured meat, this variability may constitute a significant barrier to acceptance and market entry, as differing interpretations and certification requirements can lead to regulatory fragmentation and consumer uncertainty across Muslim-majority markets. Therefore, a clear scholar’s stance enhances harmonization and mutual recognition among halal authorities, supporting consistency in global trade. Addressing these differences, whether through national frameworks or coordinated international efforts, will be essential for facilitating broader acceptance of cultured meat in Muslim societies.

This rigorous certification process is necessary due to clearly defined prohibitions. Certain foods and ingredients are strictly prohibited (haram) under Islamic law. These include pork and its derivatives, improperly slaughtered animals, intoxicants such as alcohol, blood, blood products, and any food that comes into contact with these substances [44,45]. In the modern food industry, many food ingredients are derived from animals or animal by-products, such as fats, proteins, gelatin, glycerin, hormones, and flavor enhancers [45,46,47]. Even trace contamination from haram substances renders a product non-halal, underscoring the importance of robust quality assurance systems and supply chain audits.

Given the complexity of global supply chains and varying interpretations, the regulatory leadership of certain nations becomes pivotal. Among Muslim-majority countries, the Kingdom of Saudi Arabia often plays a leading role in setting standards, particularly in areas where scientific advancements intersect with Islamic religious principles. The country’s influence stems from its religious significance as the birthplace of Islam and its efforts to ensure that modern developments remain compatible with Sharia law [48]. As a result, Saudi Arabia frequently establishes guidelines that other Muslim nations consider when evaluating the ethical and religious acceptability of new technologies, including those used in fields like biotechnology, food production, and medical research [49].

It is important to note that in many Muslim-majority countries, halal regulation operates at the intersection of religious interpretation and state-led governance. Religious scholars and fatwa-issuing bodies provide theological guidance on permissibility, which regulatory authorities then translate into enforceable certification standards. This integrated approach ensures that halal compliance is both theologically sound and systematically verifiable.

Within Saudi Arabia, the regulatory apparatus for halal is clearly delineated. The responsibility for issuing and overseeing halal certification lies with the Saudi Food and Drug Authority (SFDA) through its specialized branch, the Saudi Halal Center (SHC). The SHC ensures that food, pharmaceuticals, and cosmetics, whether imported or domestically produced, comply with Islamic dietary laws [50]. While the SFDA sets regulatory standards, the SHC accredits both domestic and international halal certification bodies. These certifiers must strictly adhere to Saudi Arabia’s halal requirements to be officially recognized [51]. For instance, any foreign company willing to export halal-certified products to Saudi Arabia must obtain certification from a body approved and listed by the SFDA.

The emergence of novel foods like cultured meat presents a contemporary challenge to this established framework, prompting activity but not yet consensus. Various Islamic organizations and halal certification bodies began addressing the halal status of cultured meat. The Malaysian Halal Certification Authority (JAKIM) and the Islamic Food and Nutrition Council of America (IFANCA) have acknowledged the need for clear guidelines on cultured meat [38]. Some Islamic scholars have issued preliminary fatwas stating that if cultured meat is derived from halal sources and remains free of haram substances, it could be permissible [42]. The Organization of Islamic Cooperation (OIC) is working on a standardized halal certification process for novel food technologies, including cultured meat. This landscape of ongoing evaluations highlights a critical point: a consensus among major Islamic authorities remains lacking, and divergent views across countries may pose significant challenges to international commercialization.

Synthesizing the current Saudi position within this global discussion reveals a cautious, review-based approach. Currently, cultured meat has not been introduced into the Saudi Arabian market—neither the SFDA nor SHC has issued halal certification or market authorization for cultured meat products. Due to ongoing theological and technical considerations, the halal status of cultured meat remains under active review by religious scholars and regulatory authorities, including the SFDA. Nevertheless, the potential of cultured meat to contribute to national food security and innovation aligns with the strategic objectives outlined in Saudi Arabia’s Vision 2030 [52].

## 3. Cultured Meat Production from a Halal Perspective

Cultured meat represents a novel approach among meat alternatives. The backbone of the technology involves in vitro cell culture, enabling the production of selected meat cuts with minimal or no animal sacrifice. Starter cells comprise embryonic stem cells (ESCs), induced pluripotent cells (iPSCs), and primary cells. ESCs are derived from an embryo, whereas iPSCs are generated by the genetic reprogramming of adult somatic cells. In turn, primary cells are retrieved through biopsy of a living animal or from the tissue of slaughtered animals. Alternatively, from fertilized eggs in the case of avian and fish species [53,54,55]. In general, progenitor and non-engineered cells are utilized in cell culture for food purposes due to public skepticism toward genetic engineering, although genetic modifications might accelerate meat production processes [53,56]. Immortalized primary cells exhibit an unlimited capacity, similar to that of ESCs. Obtaining an immortalized cell line can be spontaneous or achieved through genetic modification. And the former, spontaneous immortalization was indicated to be feasible and safe for cell culture while exponentially extending population doublings beyond 600 [55].

The cell source poses a challenge to establishing the halal status of cultured meat. Since cultured meat does not involve the direct slaughter of an animal, scholars are actively debating whether the initial cell extraction from a biopsy sample (originating from a living animal) and subsequent culture in the lab fulfill these requirements [57,58]. It is important to distinguish between the scientific feasibility of using various cell types and their religious permissibility. Some interpretations hold that a biopsy from a living animal yields material considered a carcass (haram), except for aquatic animals [42]. Conversely, others argue that isolated cells are distinct from flesh and may not be subject to the same ruling. Regarding ESCs, certain Islamic scholarly perspectives categorize them not as a body (Jism) but as a piece of blood in the uterus, meaning the female animal’s slaughtering status applies [42]. However, this interpretation is not universally agreed upon, and the permissibility of using ESCs from a halal-slaughtered animal remains a subject of ongoing scholarly debate. Similarly, the use of immortalized cell lines derived from primary cells introduces further theological complexity regarding the continuity of the original animal’s status across generations of cell division. The traditional halal certification process requires slaughtering animals by Muslims while invoking the name of Allah (Tasmiyah) [59], a requirement that is fundamentally absent in a biopsy-based process, creating a key point of contention.

Conducting in vitro cell culture requires recreating the in vivo stem cell environment by providing culturing media and bioreactors [53]. The latter mimics physical properties such as temperature or pH. Cell media consists of basal medium, serum (or serum replacements), and other additives. Basal medium mimics body fluids and contains amino acids, carbohydrates, lipids, vitamins, inorganic salts, and trace minerals [60,61]. Serum (or serum replacements) stimulates cell culture, owing to growth factors, interleukins, albumin, and other elements [20,60]. Microcarriers and scaffolds enhance cell culture, allowing the expansion of adherent cells and mimicking conventional meat morphology, which is essential for achieving a meat-like texture, a key for most consumers [62,63].

Despite promoting cultured meat as a means to minimize animal exploitation, many protocols still rely on animal-derived components [53]. Fetal bovine serum (FBS), retrieved from the fetal blood of pregnant cows, constitutes a primary component of media for cell culture [62,64]. According to Islamic principles, blood is impure, thus prohibited (Haram), which poses a significant theological barrier to the use of FBS as it is a blood component. Other animal-derived components include growth factors and extracellular matrix proteins—commonly used in media, also as substrates of microcarriers and scaffolds. The source of them should not originate from haram animals (e.g., swine, dogs, rats, or insects). For example, bovine collagen is permitted, but porcine collagen is not [42,65]. This creates a clear technical challenge: achieving scientific feasibility with animal-derived components may conflict with religious permissibility. In general, the cultured meat industry aims to exclude animal-derived components, which predominantly involves the use of recombinant proteins produced by genetically modified microorganisms [66]. From a halal perspective, this shift may offer a solution, but it introduces new questions. Within Islamic law, genetically modified organisms are generally considered halal if they are safe for human health and do not contain genes from haram species [67]. However, the permissibility of the final product when derived via microbial fermentation of recombinant proteins is an area requiring specific religious guidance. Recent analyses highlight that achieving both commercial viability and halal compliance will depend on developing affordable, scalable, and animal-free culture media, a major focus of current research [68].

## 4. Market Acceptance and Consumer Perspective

Various socio-economic and psychological factors, including price sensitivity, product availability, religious trust, and cultural perceptions, influence Muslim consumers’ market acceptance of cultured meat. However, the relative importance of these factors differs markedly across cultural, regional, and socioeconomic contexts. Affordability remains a key factor, as price-conscious consumers may be hesitant to pay a premium for cultured meat, unless it offers clear benefits over conventional halal meat [69,70]. This concern is particularly pronounced in lower- and middle-income settings, whereas higher-income urban consumers may prioritize ethical or environmental attributes.

Furthermore, consumer trust in religious authorities and halal certification bodies is essential, though levels of trust and institutional influence vary between countries and diaspora communities. Companies must engage with Islamic scholars and halal certifiers to ensure compliance and gain credibility [71]. Marketing strategies that emphasize ethical, environmental, and health benefits, along with transparent labeling and endorsements from religious institutions, can influence consumer perception [72,73]. The effectiveness of such strategies is likely to depend on locally embedded religious norms and media ecosystems. Communicating the alignment of cultured meat with Islamic values, such as animal welfare, sustainability, and food security, can further resonate with religious consumers. Additionally, demographic differences exist: younger consumers are more open to trying innovative food technologies, while older generations may show resistance due to cultural and religious conservatism. These generational differences are not uniform and may intersect with education level, urban–rural divides, and exposure to global food trends. Effective consumer education campaigns that explain the science behind cultured meat, debunk misconceptions, and clarify religious rulings can help bridge this generational gap.

Across studies conducted in Muslim-majority countries and among Muslim communities, acceptance of cultivated meat appears highly variable and context-dependent, typically ranging from 17% to 56% (Table 1). Such variability is also observed in Western countries. For example, within Europe, the Netherlands, Spain, Italy, the UK, and Germany show higher openness than France, Belgium, Finland, and Poland [69,70,72,73,74,75,76,77]. Moreover, many demographic factors are known to affect such acceptability, with younger, more educated, male, urban, less religious, environmentally/ethically concerned consumers, and those with lower food-technology neophobia more willing to try or buy cultured meat [70,73,78,79,80].

The main obstacles in acceptance of cultured meat in non-Muslim-majority countries include perceived unnaturalness, disgust, food-technology risk, and doubts about safety, healthiness, taste, and price competitiveness [70,74,81,82,83]. However, in Muslim-majority regions, religious concerns, particularly the issue of halal compliance, emerge as a consistent determinant of hesitancy, with many respondents indicating they would only consider cultured meat if it is halal-certified. Notably, acceptance rates and the weight attributed to halal status differ between countries such as Malaysia, Singapore, and Arabic-speaking nations (Table 1), as well as between urban and rural populations, younger and older cohorts, and diaspora communities versus those in Muslim-majority settings. These variations underscore the need for culturally tailored communication strategies and locally embedded certification frameworks rather than a one-size-fits-all approach. However, the relative contribution of religious versus non-religious drivers should be interpreted with caution. Evidence suggests that in some cohorts, particularly younger, urban, and more globally exposed populations, general food technology neophobia, perceptions of unnaturalness, and trust in novel production methods may play a more decisive role than halal considerations per se. In such contexts, religious concerns may interact with, or even mask, broader anxieties about technological intervention in food systems rather than act as an independent determinant of rejection.

Importantly, the evidence summarized in Table 1 should be interpreted with caution, as most studies rely on self-reported intentions, hypothetical scenarios, and non-representative samples, which may overestimate real-world adoption. Future research employing mixed methods and representative sampling across diverse Muslim contexts is needed to capture the full spectrum of consumer attitudes.

**Table 1:** Summary of cultivated meat (CM) acceptance among Muslims in Muslim-majority countries and diaspora communities. This table provides a representative overview to contextualize the relevance of halal considerations; it is not a systematic review or meta-analysis. The included studies vary in design and sample representativeness and rely primarily on self-reported intentions, which may not reflect real-world adoption.

Country	Year	Design	Studied Group	Main Observations	Reference
Singapore	2023	Survey (online)	*n* = 658 (21 to 82 years)	No specific acceptance rate reported; intention to consume CM was shaped by perceived media, family members, and influencers. Halal awareness increased attention to CM messaging, suggesting conditional openness.	[84]
Malaysia	2022	Survey (online/in-person)	*n* = 102 (18 to 62 years)	44% accepted CM; 56% had doubts due to religious concerns	[85]
Malaysia	Not reported	Survey (online)	*n* = 120 (20–30 years)	32% understood CM from a halal perspective; 41% accepted CM	[86]
Singapore	2022	Qualitative with focus group discussions	*n* = 24 (22 to 69 years)	Would only consider consuming CM if halal-certified	[87]
Arabic countries	2020–2022	Survey (online)	*n* = 1025 (18 to >51 years)	17% considered CM acceptable, 44% were interested in CM, 39% revealed reluctance	[88]
United Kingdom	2020–2021	Survey (online)	*n* = 119 (≤24 to >55 years)	44% willing to try CM, 38% willing to purchase, 15% were willing to pay extra	[89]

## 5. Challenges and Recommendations

**Figure 2:**
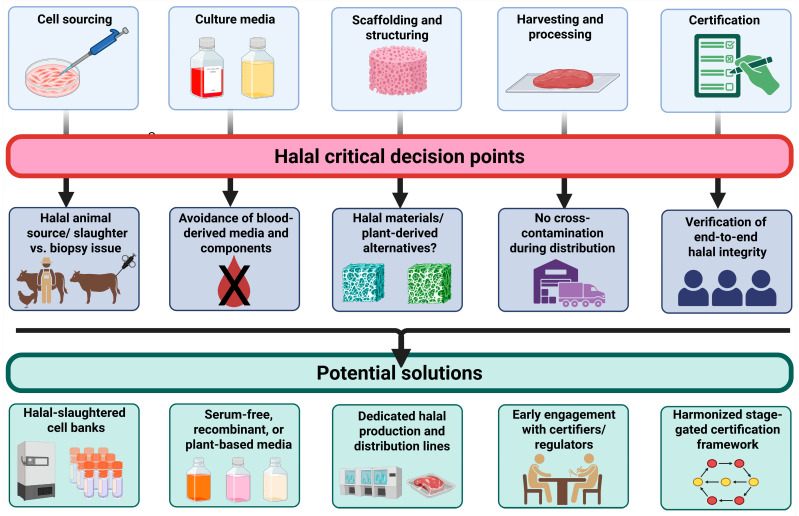
Flowchart mapping cultured meat production stages to halal critical decision points, associated risks, and proposed mitigation solutions. Graph created with BioRender.com.

Despite the apparent opportunities, the commercialization of cultured meat for Muslim consumers faces significant theological, technical, and regulatory hurdles. To navigate this complex landscape, a structured approach prioritizing key actions is essential. The primary challenges stem from the diversity of production protocols and the permissibility of components utilized. Table 2 outlines specific halal guidelines, potential mismatches, and mitigation strategies, providing a clear framework for the following prioritized recommendations. The recommendations proposed in this section are not intended as generic restatements of existing positions but as a structured, stage-specific framework aimed at translating theological debate into actionable guidance for regulators, halal certification bodies, and cultured meat developers. Given the absence of finalized or harmonized Islamic rulings on cultured meat, these recommendations should be understood as directional and facilitative rather than prescriptive. Their purpose is to identify leverage points where scientific innovation, regulatory clarification, and religious interpretation can productively converge to enable halal-compliant cultured meat systems. Importantly, the recommendations are organized along temporal and institutional dimensions—short-term (cell sourcing and media composition), medium-term (development of scalable halal-compliant inputs), and long-term (harmonization of halal certification frameworks for novel foods). This structure reflects the reality that cultured meat adoption in Muslim-majority contexts will depend not on a single religious ruling, but on coordinated progress across multiple stages of the production and governance pipeline. Figure 2 presents a flowchart that visually maps the cultured meat production stages to corresponding halal critical decision points, associated risks, and proposed mitigation strategies, providing an integrated overview of the analytical framework described in this section.

**Figure 3:**
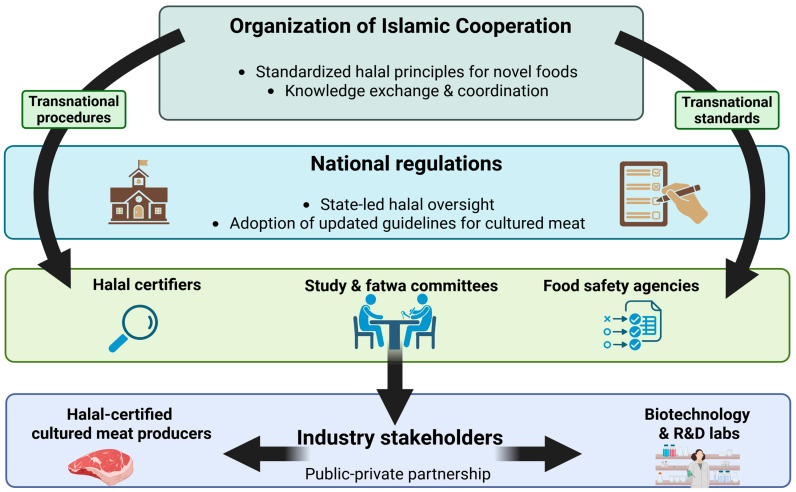
**Multi-level governance and stage-gated halal decision framework for cultured meat.** The figure illustrates how halal compliance for cultured meat emerges through coordinated decision-making across production stages (cell sourcing, culture media, processing, and certification) and governance levels (scientific development, religious jurisprudence, certification bodies, and state regulation). The framework highlights critical decision points where technological choices intersect with Islamic dietary principles, enabling the identification of regulatory leverage points and pathways toward harmonized halal certification for novel food technologies. Graph created with BioRender.com.

From a regulatory perspective, the absence of harmonized halal guidance for cultured meat suggests a need for multi-level governance mechanisms. At the transnational level, harmonization may require coordination among major halal standard-setting bodies, such as those operating within the Organization of Islamic Cooperation framework, to establish shared baseline principles for novel foods, while allowing national authorities discretion in implementation. At the national level, state-led bioeconomy strategies, particularly in food-import-dependent countries, could explicitly incorporate halal-compliant cellular agriculture as a strategic priority. Public–private partnerships involving regulators, halal certification bodies, academic institutions, and cultured meat developers may serve as testbeds for pilot halal-compliant production systems, enabling evidence-based fatwas and regulatory learning prior to full commercialization. Figure 3 conceptualizes how regulatory authorities, halal certification bodies, religious scholars, and industry stakeholders interact across governance levels to enable halal-compliant cultured meat development.

As an immediate priority, resolving uncertainties around the starting cell source is fundamental. Table 3 summarizes the major production stages, associated halal concerns, relative risk levels, and proposed mitigation strategies, to provide a structured overview of where halal compliance challenges and opportunities arise along the cultured meat value chain. This framework highlights that halal suitability is not determined by a single input or step but rather emerges from cumulative decisions made throughout cell sourcing, cultivation, processing, and certification. We recommend the preferential use of primary cells for culture rather than ESCs, since the Islamic stance on their appropriateness as food remains unknown. An exception may apply to embryos extracted from the uterus of animals slaughtered in accordance with Shariah law, in which case their status as ESCs might be considered halal [42,53]. At the same time, primary cells originating from tissue material should be obtained from a slaughtered animal rather than through biopsy from a living animal [54]. Islamic regulations allow only the consumption of mammals slaughtered according to halal guidelines, without a biopsy sample becoming a carcass. Thus, it is recommended to retrieve tissue from animals slaughtered according to halal standards [42]. However, the impact of ritual slaughter on starting material and isolated cells should be elucidated. In the avian case, fertilized eggs are commonly a source of primary cells [55,90]. Yet the developmental stage of the avian embryo at the time of extraction and whether the egg was sourced from a halal-slaughtered bird requires further religious and scientific clarification. This issue should be formally addressed by SHC and other relevant Islamic bodies.

A critical medium-term priority is the development of economically feasible, scalable, and halal-compliant production systems. Cultured meat manufacturers targeting the Muslim market must exclude from their production process materials forbidden by Islamic law, such as porcine products [91,92] or blood-derived products [60]. Although Islamic opinion on FBS utilization remains unclear, given the prohibition on consuming blood from all mammals and birds, its approval remains unlikely [93]. Precision agriculture allows the production of recombinant components that replace FBS, growth factors, and other animal-derived proteins. However, the economic feasibility and scalability of these animal-free, halal-compliant media must be a primary focus of research and development to enable commercial viability. Yet, the regulatory framework regarding recombinant proteins within Islamic law remains unspecified [66,94], and by extension, inhibits reaching Muslim consumers. Further scholarly discourse is urgently needed to determine whether recombinant products derived from microbial or plant-based expression systems (not involving haram genetic material) can be considered halal under Shariah principles. Clear and consistent fatwas or religious rulings will be essential to remove ambiguity and build consumer confidence.

For long-term market integration, establishing a unified and practical halal certification framework is essential. Food-related shifts involving novel technologies, such as cell culture for food purposes, require in-depth analysis. Consuming a particular product depends not exclusively on sensory, safety, and price factors, but also on religious beliefs that shape dietary habits. Therefore, fitness to various religious requirements should be addressed in order to disseminate new technologies in the food sector for sustainable economies. Policymakers and industry stakeholders should collaborate with religious authorities to develop clear, stage-gated guidelines that balance theological principles with practical scalability. In this context, aligning biotechnological innovation with ethical and spiritual values presents a rare opportunity not only to promote inclusivity but also to position Islamic scholars as active stakeholders in shaping the global food future.

To further operationalize these recommendations, we propose a “Halal-by-Design” (HbD) protocol tailored to cultured meat startups, analogous to quality-by-design approaches used in biopharmaceutical manufacturing. In practical terms, HbD would require that halal compliance be embedded from the earliest stages of process development rather than assessed post hoc. Such an approach reduces regulatory uncertainty, minimizes costly reformulation, and aligns product development with certification requirements from the outset.

A minimal HbD protocol would include:

- Certified sourcing of starting materials, including the establishment of traceable cell banks derived exclusively from halal-slaughtered animals under the supervision of recognized halal authorities;
- Upfront selection and documentation of all media components, scaffolds, and processing aids, ensuring either halal-certified origin or demonstrable compliance through transformation (istihalah) or synthetic/recombinant pathways;
- Implementation of segregated or validated cleaning-compliant production lines to prevent cross-contamination with non-halal inputs;
- Real-time documentation and auditability of the production process to facilitate certification review;
- Early-stage engagement with halal certification bodies to enable iterative feedback rather than end-point approval.

Equally important is the institutionalization of cooperation mechanisms to support this model. Beyond general calls for collaboration, concrete pathways include the creation of joint scientific–jurisprudential advisory boards embedded within pilot facilities, co-development agreements between startups and national halal authorities (e.g., SFDA, JAKIM), and transnational working groups under platforms such as the Organization of Islamic Cooperation to standardize evaluation criteria for novel food technologies. Embedding religious scholars within R&D pipelines, rather than consulting them only at the certification stage, may prove particularly critical for resolving ambiguities related to recombinant inputs, cell ontologies, and istihalah. These structured partnerships can function as “regulatory sandboxes,” enabling iterative testing of halal compliance in parallel with technological optimization.

The recommendations outlined above are not generic prescriptions but are derived directly from the stage-based halal compliance framework introduced in this paper. As summarized in Table 3, each production stage carries distinct halal risks that necessitate stage-specific mitigation strategies. For instance, cell sourcing risks (high) require preferential use of primary cells from halal-slaughtered animals; growth medium risks (high) demand investment in animal-free, recombinant media; and certification risks (high) call for a harmonized, stage-gated governance framework. Translating these insights into practice will require coordinated action across multiple stakeholders. Specifically, we recommend the following actionable pathways: (i) the establishment of pilot halal-compliant cell banks under the oversight of the SHC to ensure traceable, certified starting materials; (ii) the formation of a joint technical-religious working group involving the SFDA, major halal certification bodies (e.g., Department of Islamic Development Malaysia—JAKIM, Islamic Food and Nutrition Council of America—IFANCA), and cultured meat developers to harmonize certification criteria; and (iii) targeted public–private partnerships to scale the production of halal-certified, serum-free culture media. These strategies move beyond conceptual advocacy to concrete institutional mechanisms that can facilitate responsible market entry.

## 6. Conclusions

This narrative perspective article set out to systematically examine how specific stages of cultured meat production align with, or challenge, Islamic dietary principles, and to propose a stage-based analytical framework to guide halal compliance efforts. Cultured meat has transformative potential to address the pressing challenges of food security, environmental degradation, animal welfare, and antimicrobial resistance. However, realizing this potential requires more than just technological advancement; it necessitates cultural, religious, and regulatory alignment, particularly in Muslim-majority countries where halal compliance is essential to dietary acceptance. As this perspective article demonstrates, cultured meat presents both a challenge and an opportunity for halal certification frameworks. The source of cells, the composition of the growth medium, and the use of animal-derived or recombinant materials all intersect with Islamic law, requiring clear guidance and a regulatory framework.

Importantly, efforts to align cultured meat with halal standards may catalyze broader innovations in cellular agriculture, such as the development of serum-free, animal-free culture media. These innovations would not only support religious inclusion but also enhance the scalability, safety, and reproducibility of cultured meat production globally. In this sense, addressing halal requirements is not a barrier—it is a strategic inflection point that can accelerate technological refinement and market diversification.

One should note that our paper is limited by the absence of empirical data from halal-certified cultured meat production systems and by its reliance on evolving theological interpretations rather than finalized or universally accepted religious rulings. Addressing these gaps requires a more explicit and time-bound research agenda. Over the upcoming years, three priorities are particularly critical. First, there is a need for empirical, laboratory-based studies examining the istihalah (transformation) status of specific production inputs (especially scaffolding materials, recombinant growth factors, and culture media residues) under conditions relevant to industrial processes. Second, structured collaboration between scientists, Islamic scholars, and halal certification bodies is required to generate evidence-informed, consensus-oriented guidance on contentious issues such as cell sourcing (including stem cells) and the permissibility of recombinant or synthetic components. Third, large-scale, representative consumer studies in major urban centers in the MENA region should move beyond hypothetical acceptance to assess responses to certified or near-market cultured meat prototypes, including willingness to purchase and trust in certification systems.

These priorities are inherently sequential. Foundational work—empirical validation of halal compliance at the material level and jurisprudential clarification—must precede downstream efforts such as techno-economic modeling and consumer adoption studies. Without resolving these upstream uncertainties, subsequent analyses risk remaining speculative and of limited policy relevance.

Finally, proactive engagement with Islamic scholars, certification authorities, and Muslim communities will be essential to ensure legitimacy and public trust. Transparent communication and inclusive governance can support informed acceptance and enable Muslim-majority countries to play an active role in shaping the future of cellular agriculture. By bridging advances in food biotechnology with deeply rooted ethical and religious frameworks, cultured meat has the potential to emerge as a genuinely inclusive innovation aligned with both sustainability goals and societal values.

## Figures and Tables

**Table 2:** Halal guidelines for meat in the context of compatibility with the cultured meat concept and mitigation strategies for potential mismatch.

Halal Guideline	Description	Cultivated Meat Status	Potential Mismatch	Possible Mitigation Strategy
The source animal must be permissible (Halal)	The meat must come from animals permitted in Islam (e.g., cow, chicken, sheep). Swine and carnivores are forbidden.	Cultivated meat is usually derived from permitted animals, but cells may also be taken from forbidden species in some experiments.	Possible mismatch if cultivated from haram species (e.g., swine).	Ensure cell lines are only sourced from halal animals and communicate this transparently.
The animal must be alive at the time of slaughter	Islamic law requires animals to be alive when slaughtered.	Cultivated meat is produced from a cell biopsy; no whole animal is slaughtered.	Conceptual mismatch—no slaughter occurs.	Scholars may consider this as not requiring slaughter if no sentient life exists; they seek fatwas based on analogy (Qiyas) and expert consensus (Ijma).
Proper Islamic slaughter (Thabiha)	Includes invoking Allah’s name (Bismillah), blood drainage, and a specific method of cutting.	No blood, no slaughter in a conventional sense.	Slaughter rituals cannot be performed.	If the initial cell donor animal was slaughtered Islamically, some scholars may consider the process acceptable. Alternatively, separate cell banks based on cells retrieved from the tissues of ritually slaughtered animals. Dialogue with Islamic jurists is key.
No contamination with haram substances	No use of alcohol, porcine enzymes, pork derivatives, or non-halal ingredients in processing.	Cultivation may involve fetal bovine serum (FBS), animal-origin nutrients, or porcine-derived components.	High mismatch if FBS or haram additives are used.	Develop fully plant-based or halal-certified growth media. Ensure contamination control.
Processing tools must be halal-compliant	Equipment must be clean and free from contact with haram substances.	Manufacturing equipment could be shared with non-halal products.	Risk of contamination.	Certify dedicated halal production lines or apply strict cleaning protocols and certification standards.
Ethical treatment of animals	Islam emphasizes kindness and minimizing animal suffering.	Cultivated meat avoids raising and killing animals.	No mismatch—rather, an ethical advantage.	Highlight alignment with Islamic ethical values to support acceptance.

**Table 3:** Stage-specific halal risks and mitigation strategies in cultured meat production.

Stage	Halal Concern	Risk Level	Suggested Solution
Cell source	ESCs, biopsies from live animals	High	Primary cells from halal-slaughtered animals
Growth medium	FBS, blood-derived components	High	Plant-based or recombinant halal-certified media
Processing	Shared equipment, contamination	Medium	Dedicated halal lines, certified cleaning protocols
Ethical dimension	Animal welfare	Low	Alignment with Islamic ethical principles
Certification	Lack of unified fatwas	High	Stage-gated halal framework with SHC involvement

ESCs—embryonic stem cells; FBS—fetal bovine serum; SHC—Saudi Halal Center.

## Data Availability

The original contributions presented in this study are included in the article. Further inquiries can be directed to the corresponding author.
